# Is Heel Height Associated with Pain Exacerbations in Hip Osteoarthritis Patients?—Results from a Case-Crossover Study

**DOI:** 10.3390/jcm9061872

**Published:** 2020-06-16

**Authors:** Kai Fu, Ben R. Metcalf, Kim L. Bennell, Yuqing Zhang, K. Douglas Gross, Kathryn Mills, Leticia A. Deveza, Sarah R. Robbins, David J. Hunter

**Affiliations:** 1 Kolling Institute of Medical Research, Institute of Bone and Joint Research, University of Sydney, Sydney, NSW 2065, Australia; leticia.alle@sydney.edu.au (L.A.D.); sarah.robbins@sydney.edu.au (S.R.R.); david.hunter@sydney.edu.au (D.J.H.); 2 Department of Rheumatology, Royal North Shore Hospital and Northern Clinical School, University of Sydney, Sydney, NSW 2065, Australia; 3 Centre for Health, Exercise and Sports Medicine, Department of Physiotherapy, University of Melbourne, Melbourne, VIC 3053, Australia; b.metcalf@unimelb.edu.au (B.R.M.); k.bennell@unimelb.edu.au (K.L.B.); 4 Division of Rheumatology, Allergy, and Immunology, Massachusetts General Hospital, Harvard School of Medicine, Boston, MA 02115, USA; yzhang108@mgh.harvard.edu; 5 Department of Physical Therapy, MGH Institute of Health Professions, Boston, MA 02129, USA; dgross1@mghihp.edu; 6 Department of Health Professions, Macquarie University, Sydney, NSW 2109, Australia; kathryn.mills@mq.edu.au

**Keywords:** osteoarthritis, hip, pain, footwear, case-crossover study

## Abstract

The etiology of osteoarthritis (OA) pain exacerbations is not well understood. The purpose of this study is to evaluate the association of heel height and duration of wearing shoes with higher heels with pain exacerbations in people with hip OA. Eligible participants with symptomatic hip OA were instructed to complete online questionnaires every 10 days over a 90-day follow-up period. They were required to complete the questionnaire whenever they were experiencing hip pain exacerbation. Of 252 participants recruited, 137 (54.4%) contributed both case and control period data, and were included in the analysis. Wearing shoes with a heel height ≥ 2.5 cm during the past 24 h was associated with lower odds of pain exacerbations (OR: 0.54, 95% CI: 0.30 to 0.99). A longer duration (>6 h) of wearing shoes with heel height ≥ 2.5 cm was also associated with a lower risk of hip pain exacerbations (*p* for linear trend = 0.003). Wearing shoes with heel height ≥ 2.5 cm and longer duration in the past 24 h may be protective against hip pain exacerbations in people with symptomatic hip OA. Given the observational study nature, it would be prudent for this to be replicated in an independent data set.

## 1. Introduction

Osteoarthritis (OA) of the hip is the second most common site of lower limb OA after the knee, with an estimated overall prevalence in the general adult population of 11%, and somewhat higher in older adults [1,2]. 

Pain is the ubiquitous symptom of OA and a major driver of clinical decision-making. While patients with OA often have persistent pain [3], many experience pain fluctuations or periods of exacerbation during which pain and functional impairment worsen [4]. In the early stages of OA, pain is often intermittent, becoming more frequent and severe as the disease progresses [5]. The etiology of OA pain exacerbations is not well understood, despite recent progress in the understanding of chronic OA pain [6].

As disease-modifying treatment is not yet available for OA, pain-modifying treatments remain essential [7]. Key treatments include education, exercise, weight loss when needed and walking aids, as indicated [7]. Appropriate footwear may be a cost-effective and safe intervention for managing the painful symptoms of hip OA [8]. Though people regularly wear high-heeled shoes, it has been suggested that continued utilization may negatively influence lower extremity musculoskeletal health [9]. But the mechanism behind this has not been fully elucidated. There are many studies that have measured the biomechanical effects of shoes with higher heels in people with knee OA, but not hip OA, showing that wearing heels of increasing height could increase the medial tibiofemoral compartment and patellofemoral joint loading [9]. Furthermore, there is a strong association between medial knee load and increased pain during walking in patients with knee OA [10]. Nonetheless, these results cannot necessarily be generalized from knee OA to hip OA, given differences in risk factors, biomechanics and response to treatment [11].

To our knowledge, no study has examined the effect of footwear on pain exacerbations in people with hip OA. To better understand this, we conducted an Internet-based, case-crossover study to evaluate the associations of wearing shoes with higher heels and the duration of time spent wearing shoes with higher heels with hip pain exacerbations in people with symptomatic hip OA. Our hypothesis was that higher-heeled shoes, especially if worn for a longer duration, would be associated with a greater risk of pain exacerbations in people with hip OA. 

## 2. Experimental Section

### 2.1. Study Design

An Internet-based, case-crossover study was designed to assess the relation of wearing shoes with different heel heights, and the duration of wearing such shoes to the risk of hip pain exacerbation in Australia from May 2015 to June 2017. This design has previously been described in hip and knee OA pain exacerbation studies [12,13,14,15,16,17]. Briefly, the case-crossover design uses each participant as their own control to assess the effects of transient exposures (risk factors) on episodic events (e.g., pain exacerbation) during a certain follow-up period (e.g., 90 days). The study was approved by the ethics committees of the University of Sydney (HREC 2014/801) and University of Melbourne (HREC 1443509), and all participants provided informed consent.

### 2.2. Study Population 

To be eligible for the study, participants were required to: be ≥40 years old; self-report hip pain on most days (5–7 days/week or 20–30 days/month); have at least one hip meeting American College of Rheumatology radiological criterion for hip OA [18]; have a Kellgren and Lawrence grade of hip OA ≥ 2 [19]; have access to the Internet; and have a good understanding of spoken and written English. Participants were excluded if they had a history of total hip replacement in the index hip, a scheduled total hip replacement or a consultation with an orthopedic surgeon for consideration of total hip replacement of the symptomatic hip(s); or a history of inflammatory arthritis, osteonecrosis or Paget’s disease affecting the hip.

### 2.3. Data Collection

An online screening survey tool was designed to recruit study participants across Australia. We advertised the study through social media (Facebook), patient advocacy websites (Arthritis Australia, etc.), local newspapers and flyers.

When a potential study candidate registered interest in participation, their contact details were emailed to a study coordinator (in Sydney or Melbourne, depending on their state). The study coordinators then contacted participants for further assessment and enrollment. Meanwhile, the participants were also asked to post their most recent X-rays (within one year) to the study coordinators for eligibility criteria assessment. Radiographic grading of hip OA was read by two rheumatologists (L.A.D. and D.J.H.) in our study. The inter-rater reliability was assessed using 12 radiographs providing a weighted kappa of 0.6 for Kellgren and Lawrence grades. Eligible participants were enrolled and provided access to the study’s website. Participants were followed for 90 days and asked to complete online questionnaires at baseline and every succeeding 10-day interval. The intensity of hip pain was assessed using an 11-point numeric rating scale (NRS; ranging from 0—“no pain” to 10—“worst pain possible”), which showed high test-retest reliability in osteoarthritis populations [20,21]. Participants were asked to indicate how bad their hip pain was at its mildest and worst in the baseline questionnaires. Consistent with previous studies [12,13,14,15,16], a pain exacerbation was operationally defined as an increase of ≥2 points in the participant’s pain level compared with their mildest hip pain level reported in the baseline questionnaire. We used the mildest pain level at baseline as the comparator to facilitate the identification of the maximum number of pain exacerbation events [13]. When a participant considered that they were experiencing a pain exacerbation, they were asked to complete an online questionnaire, which automatically determined whether it met the operational definition of a pain exacerbation by comparing their current reported pain level with the baseline pain level. If so, they were then guided to complete the questionnaire. To avoid biased reporting, the amount of pain increase needed to be defined as a pain exacerbation was not disclosed to participants. The system sent reminder emails during every 10-day interval to remind the participants to log onto the study website when they were experiencing any pain exacerbations. A case period was defined as a 24 h period before a pain exacerbation report and a control period was a 24 h period before every 10-day interval report. A case or control period was marked as missing if questionnaire data were not entered within 48 h when there was a pain exacerbation reported. Risk factor assessment questionnaires for control periods and the case periods were the same for all online visits.

Shoes were categorized into three different styles based on heel height (<2.5 cm or 1 inch; 2.5–5 cm or 1–2 inches; >5 cm or 2 inches). Participants were asked to report which type(s) of shoes, including the heel height, they wore during the previous 24 h, and how long they were worn. To facilitate more accurate reporting of shoe heel height, participants could choose from a list of different types of shoes on the study website. The questionnaire was derived from that used in the population-based Framingham Foot Study [22,23]. We also used the International Physical Activity Questionnaire (IPAQ) short form to evaluate physical activity during the previous seven days, as physical activity could influence the choice of shoe and OA pain [24,25]. The IPAQ short form assesses the types of intensity of physical activity and sitting time that people do as part of their daily lives, which are considered to estimate total physical activity in Metabolic Equivalent of Tasks (METs)—min/week and time spent sitting. The reliability and validity tests suggest that these measures have acceptable measurement properties [26]. Three levels of physical activity (1. low, 2. moderate, 3. high) based on this questionnaire were used to classify participants.

### 2.4. Statistical Methods

Baseline characteristics were summarized as mean (standard deviation, SD) for continuous variables and frequency (%) of each response for categorical variables. Characteristics were summarized for all participants enrolled in the study. However, participants who did not provide data on both case periods, and control periods were excluded from the subsequent analysis. Independent sample *t*-tests, chi-square tests and nonparametric tests (Wilcoxon rank-sum test) were performed to compare those participants with both case/control periods and those without. The analyses pooled recurrent event (pain exacerbations) data under the assumption that the within-participant correlation was accounted for by conditioning on participant-specific variables (observed or unobserved), and observed time-varying factors. 

**Figure 1:**
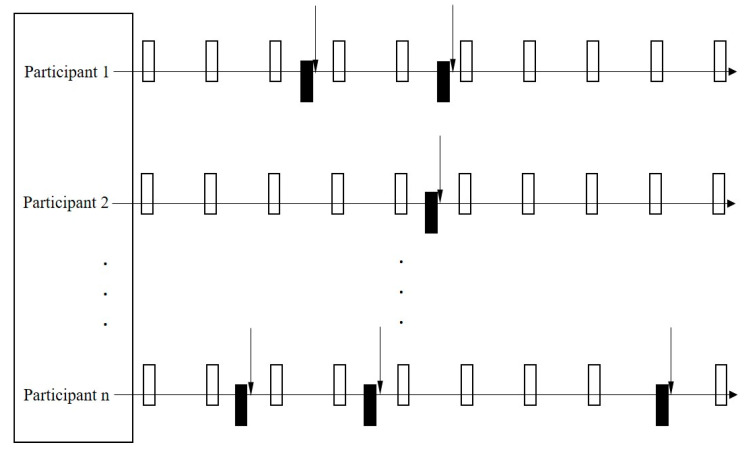
Case crossover study design (m:n matched). Each participant was followed up for 90 days. 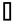 represents every control period; 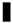 represents every case period; 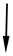 represents every pain exacerbation report time point.

Any time overlap between the case and control periods was avoided. When a pain exacerbation occurred on the same day of a normal 10-day interval report, the previous 24 h period was marked as a case period but not a control period. As each participant could contribute multiple case and control periods, an m:n matched case-control study design was used to assess the association (Figure 1). The associations of wearing shoes with higher heel heights, and the time of high-heel wearing over a 24-hour period with hip pain exacerbation, were assessed using separate conditional logistic regression models and adjusted for physical activity level. A cut-off of 6 h of wearing a heel height ≥ 2.5 cm in the past 24 h was chosen arbitrarily to differentiate those who wore them for a relatively long time. Odds ratios (ORs) were reported with corresponding 95% confidence intervals (CIs). Post-hoc analyses were also conducted to assess the interaction between the intensity of pain (at its mildest) measured at baseline, and the risk of pain exacerbation associated with heel height and the duration of wearing shoes with higher heels. Analyses were conducted using STATA version 15 (StataCorp LLC., College Station, TX, USA).

## 3. Results

Among 252 participants recruited, 137 (54.4%) had at least one episode of pain exacerbation (and at least one control period), and were included in the analysis. The majority were female (114, 83.2%) and Caucasian (130, 95%). On average, these participants were 62.6 (SD: 9.8) years old with a self-reported body mass index (BMI) of 29.0 (6.3) kg/m^2^. More than 60% of the participants had received a tertiary education (higher than high school education) and 78.8% performed light physical work (sedentary work or standing occupation). More than half of the participants had a Kellgren and Lawrence grade 3 hip OA. The baseline mean (SD) mildest and worst pain levels were 2.5 (2.0) out of 10 and 8.0 (1.7), respectively. Participants included in the final analysis (*n* = 137) showed a higher baseline pain intensity (at worst) compared with those excluded (*n* = 115) (*t*-test *p* < 0.001) (Table 1).

There was only a small proportion of periods where participants reported wearing shoes with heels > 5 cm (10 case and 30 control periods, both less than 5%). As a result, we combined heel heights > 5 cm with 2.5–5 cm in the analysis. 

In univariable conditional logistic regression analysis, wearing shoes with heel height ≥ 2.5 cm during the past 24 h was significantly associated with a decreased odds of pain exacerbations (heel height ≥ 2.5 cm vs. heel height < 2.5 cm: OR: 0.54, 95% CI: 0.30 to 0.99). There is no substantial difference after adjusting for physical activity level in the past 7 days (OR: 0.59; 95% CI: 0.32 to 1.08), and the effect estimates remained similar (Table 2).

A longer duration of time wearing shoes with heel height ≥ 2.5 cm during the past 24 h was associated with a lower risk of hip pain exacerbations. The odds ratios were 1.0 (reference), 0.94 and 0.28, respectively, for wearing times of 0 h, 0–6 h and >6 h (*p* for linear trend = 0.003) (Table 3). This remained significant after adjusting for physical activity level in the past 7 days.

The post hoc interaction analyses did not show any effect of baseline pain level (at its mildest) and risk of pain exacerbation with heel height or the wearing time (*p* values > 0.05).

## 4. Discussion

We found that wearing shoes with a heel height ≥ 2.5 cm in the past 24 h, compared with a heel height < 2.5 cm, was inversely associated with hip pain exacerbation in persons with symptomatic hip OA. This association remained even after adjusting for differences in physical activity during the previous week. There was also an independent dose—response relationship, wherein the longer the person wore shoes with a heel height ≥ 2.5 cm during the past 24 h, the lower the risk of pain exacerbation. 

To our knowledge, there is no previous study investigating the relationship between shoe heel height and symptoms in people with hip OA. A recent systematic review of the effects of footwear on joint pain and function in older adults with lower limb OA found a limited number of randomized control trials evaluating wedge insoles, shock-absorbing insoles and hardness of shoe soles, but none specifically relating to heel height [27]. 

Our study failed to confirm our hypotheses, and surprisingly found a converse relationship, which seems somewhat counter-intuitive based on results of studies in women without OA. In this population, wearing shoes with higher heels has numerous biomechanical and neuromuscular effects on the lower limb, including the hip and pelvis, which could appear detrimental to the musculoskeletal system. In the frontal plane, a significant increase in the hip abduction moment after heel strike was found from walking in a high-heel condition (up to 9 cm) compared with barefoot walking [28,29]. In the sagittal plane, a prolonged hip flexion moment was found in high-heel conditions during the late stance phase which resulted in a 23% increase in concentric hip flexor muscle work [30]. Hip angles were larger during the stance phase in the high-heel condition [31]. Increased lumbar erector spinae muscle activity and lower pelvic range of motion were also found to be associated with wearing high heels [32]. Nonetheless, effects of higher heel shoes in women without OA may differ to those with hip OA. Furthermore, the high-heel shoes tested were much higher than those worn by the participants in our study.

Gait adaptations in people with hip OA might provide one plausible mechanism by which wearing shoes with a higher heel might be associated with a lower risk of hip pain exacerbation [31,33]. People with hip OA show gait adaptations that may be strategies to avoid pain and decrease hip joint loads, and/or result from a limitation in passive motion, particularly hip flexion contracture [33]. Possible ways to compensate for inadequate hip extension are to increase lumbar lordosis and tilt the pelvis anteriorly, strategies which can occur via wearing higher heels [34]. Thus, wearing shoes with higher heels (up to a point) might indirectly help to compensate for inadequate hip extension, decreasing pain and leading to fewer pain exacerbations. However, an important caveat to this theory is that there is significant variability between studies, with many reporting that high-heeled (>4 cm) walking showed a decrease or no change in lumbar lordosis [35,36]. 

We should acknowledge that another possible reason for these counter-intuitive results, is that wearing shoes with high heels means that people spend less time walking around or doing intense activities which could lead to less likelihood of having pain exacerbations by avoiding aggravating the hip joint. In our study, the inverse association between heel height and hip pain exacerbations remained comparable even after adjusting for differences in physical activity during the previous week. People with OA have higher pain levels and fatigue symptoms and decreased physical activity when compared with controls, and fatigue had a strong negative relationship to physical activity [37]. High heel wearing could result in compensatory changes, such as increased lower limb muscle activity [28], which contributes to the higher energy cost when walking in high heels. Our previous study showed that fatigue was associated with pain exacerbations in persons with symptomatic hip OA [17], and this implies a potential indirect association between physical activity level with pain exacerbations through fatigue. What should be noted is that most people, especially women who wear high heels on a regular basis, spend the majority of time sitting rather than walking, which might contribute to a lower physical activity level [36]. In our study, we found that the effect of heel height on pain exacerbations became smaller, which can be explained by the reduced physical activity and less potential fatigue when wearing relatively higher heels. This might also explain the association between the duration of high heel wearing and pain exacerbations in our study.

Our study has limitations that should be acknowledged. Firstly, the assessment of shoe heel height was crude and relied on self-report. We did not assess the heel height relative to the ground or relative to the forefoot, and the base of the heel in terms of its width (wide-based vs. narrow-based heel), which could influence gait biomechanics. Secondly, a very small proportion of participants reported wearing shoes with heel heights > 5 cm. Thus, we cannot make conclusions about the association of wearing high-heeled shoes on pain exacerbations in hip OA, and it may be that the associations differ from those with shoe heel heights that are less extreme. Thirdly, the analyses do not take into account the shoe-wearing habits of individuals (such as whether they are a frequent wearer of shoes with higher heels), as an adaptation to footwear type might influence the relationship. There is potential confounding by indication, which might lead to distorted associations, as we did not have data before and on the day they wore higher shoes. Moreover, this relationship could be confounded by some other time-varying variables that we have not measured. Although the effect estimate remained similar after adjusting for physical activity level, we cannot confirm the role (confounder or mediator) of physical activity in this relationship based on the current analyses we have done.

Although we found these results in our study, we are not able to recommend people with hip OA to wear high heels up to 5 cm for longer periods, as this might lead to more severe constant pain and other issues related to foot, knee, low back and neck. Further research is warranted to clearly determine the advantageous heel height point before more definitive conclusions can be drawn.

## 5. Conclusions

Although we have limited data relating to wearing high heels > 5 cm, our results suggest that heels up to 5 cm could be worn with less probability of hip pain exacerbations in people with symptomatic hip OA. A longer time of wearing shoes with heels ≥ 2.5 cm in the past 24 h was also inversely associated with hip pain exacerbations. By applying a case-crossover design, all the confounding by constant characteristics such as age, sex, BMI, etc. can be eliminated during a 90-day follow-up by the pairing of cases to themselves. The findings of this study need to be corroborated in additional investigations before definitive recommendations about footwear for this patient group can be made.

## Figures and Tables

**Table 1:** Baseline characteristics of participants.

Characteristics (Mean (SD or %))	Participants Included (*n* = 137)	Participants Excluded (*n* = 115)
Age (years)	62.6 (9.8)	61.8 (8.3)
Female, *n* (%)	114 (83.2%)	85 (73.9%)
BMI (kg/m^2^)	29.0 (6.3)	28.3 (5.8)
Index hip (right)	74 (54%)	69 (60%)
Race		
Caucasian	130 (95%)	112 (97.4%)
Others	7 (5%)	3 (2.6%)
Education level		
Less than high school	22 (16.1%)	15 (13%)
Completed high school	28 (20.4%)	30 (26.1%)
Higher than high school	87 (63.5%)	70 (60.9%)
Occupational physical workload level		
Sedentary (mostly sitting)	58 (42.3%)	53 (46.1%)
Standing occupation, physically light	50 (36.5%)	40 (34.8%)
Manual work	26 (19%)	21 (18.3%)
Heavy manual work	3 (2.2%)	1 (0.9%)
Kellgren and Lawrence grade		
2	48 (35.0%)	36 (31.3%)
3	79 (57.7%)	63 (54.8%)
4	10 (7.3%)	16 (13.9%)
Baseline pain level (0–10)		
Mildest	2.5 (2.0)	2.0 (1.7)
Worst *	8.0 (1.7)	7.1 (2.0)
IPAQ (1–3); median (IQR)	3 (2)	3 (2)

* Independent sample *t*-test (*p* < 0.001). IPAQ: International Physical Activity Questionnaire; IQR: Interquartile range.

**Table 2:** Association between wearing shoes with heel height ≥ 2.5 cm (yes vs. no) over the past 24 h and pain exacerbation (*n* = 137).

Heel Height ≥ 2.5 cm	Control Periods	Case Periods	OR (95% CI)	*p* Values	Adjusted OR (95% CI) *	*p* Values
No	798 (84%)	308 (87%)	1.0 (Reference)	0.046	1.0 (Reference)	0.090
Yes	153 (16%)	46 (13%)	0.54 (0.30, 0.99)		0.59 (0.32, 1.08)	

* Adjusted for average physical activity level in the past 7 days.

**Table 3:** Association between duration of time wearing shoes with a heel height ≥ 2.5 cm over the past 24 h and pain exacerbation (*n* = 137).

Duration of Wear Time	Control Periods	Case Periods	OR (95% CI)	*p* for Trend	Adjusted OR (95% CI) *	*p* for Trend
0 h	798 (84%)	308 (87%)	1.0 (Reference)	0.003	1.0 (Reference)	0.006
0–6 h	52 (6%)	20 (6%)	0.94 (0.47, 1.88)		1.03 (0.50, 2.10)	
>6 h	101 (10%)	26 (7%)	0.28 (0.12, 0.64)		0.31 (0.13, 0.71)	

* Adjusted for average physical activity level in the past 7 days.
